# Establishing consistency and best practice in the care of children and adolescents with eating disorders: developments at the Mater Children's Hospital (MCH) and Mater Child and Youth Mental Health Service (CYMHS)

**DOI:** 10.1186/2050-2974-1-S1-O61

**Published:** 2013-11-14

**Authors:** Kelly Lamb, Suzi Scholey

**Affiliations:** 1 Mater Child and Youth Mental Health Service, Australia; 2 Headspace Gold Coast, Australia

## 

One of the biggest challenges for an organisation and for clinicians and practitioners is implementing a new program or a new practice. Following an 18 month research project and the recruitment of an Eating Disorders Coordinator the MCH and Mater CYMHS have developed and implemented a number of protocols across its service to ensure consistency in the continuum of care and to ensure that treatment is in line with the best current evidence base.

Within the campus based services these have included;

• Development of a protocol for the CYMHS Inpatient Unit to provide structure and consistency to the admission of patients with restrictive eating disorders.

• Design and roll out of a Care Pathway for the Paediatric Medical Ward where medical crises associated with malnutrition are managed.

• Guidelines for triage of such patients in the Children's Emergency Department with parameters indicative of further consultation and/or admission.

• Off campus/outpatient service development has included;

• The implementation of Maudsley Family Based Therapy as the model of choice giving structure and consistency to outpatient treatment.

• Guidelines for community staff for medical monitoring nutritionally compromised children and adolescents.

This presentation will refer to implementation science as a framework to describe the process and stages of development involved in the implementation of these projects.

This abstract was presented in the **Children and Youth Treatment and Service Development** stream of the 2013 ANZAED Conference.

